# Pure red cell aplasia due to antibody against erythropoietin in hemodialysis patients

**DOI:** 10.15171/jnp.2017.05

**Published:** 2016-08-22

**Authors:** Maryam Rahbar, Zahra Chitsazian, Firoozeh Abdoli, Seyed-Masoud Moeini Taba, Hosein Akbari

**Affiliations:** ^1^Department of Nephrology, Tehran University of Medical Sciences, Tehran, Iran; ^2^Department of Internal Medicine, Kashan University of Medical Sciences, Kashan, Iran; ^3^Departement of Biostatics and Epidemiology, School of Public Health, Kashan University of Medical Sciences, Kashan, Iran

**Keywords:** Pure red cell aplasia, Anti-erythropoietin antibodies, Chronic kidney disease, Hemodialysis, Erythropoietin

## Abstract

**Background:**

Anemia is a common complication of chronic renal failure due to reduce erythropoietin production by kidneys. Anemia treated with recombinant human erythropoietin (rHu-EPO). Pure red cell aplasia (PRCA) due to antibody productionagainst rHu-EPO is a rare but major complication of this drug.

**Objectives:**

The aim of this study was to determine the prevalence of PRCA due to antibodies in dialysis patients with resistant anemia who received erythropoietin.

**Patients and Methods:**

We studied 128 under maintenance hemodialysis patients more than 3 month in Kashan. In patients with anemia who received erythropoietin with dose requirements based on weight and anemia and without any another cause for anemia, evaluate for PRCA and anti-rHu-EPO antibody level were measured by ELISA.

**Results:**

In this research, 75 patients (58.6%) were male and 53 patients (41.4%) were female. The mean age of the patients was 59.05 ± 16.66 years. The result of analysis showed that 55 (43%) patients had anemia with hemoglobin level less than 10 mg/dL. Only 3 patients had PRCA and antibodies against erythropoietin in serum. There were no correlation between age, gender, cause of renal failure, hemodialysis duration, hemoglobin level, rHu-EPO dose and levels of anti-rHu-EPO antibody serum value.

**Conclusions:**

The result of this study indicated that administration of rHu-EPO in dialysis patients afflicted to kidney failure may cause PRCA especially through intravenous injection. However, this change is not statistically significant.

**Implication for health policy/practice/research/medical education:**

Administration of erythropoietin in patients suffering from kidney failure particularly through the venous route increases the incidence of red blood cell aplasia. However, this increase was not statistically significant and this condition may occur due to any of the two methods venous or subcutaneous injection.

## 1. Background

Chronic kidney disease (CKD) is a pathologic process that is caused by different factors (1). Anemia is one of the CKD complications that is associated with weakness and fatigue, decreases of physical and mental activities, left ventricular hypertrophy, angina, heart failure and increases cardiac mortality (1,2). Decrease immunity and defense against infection and growth retardation in children is also complications of this problem (3).

The anemia of CKD is due to many factors including decrease in erythropoietin secretion, iron, folic acid and vitamin B12 deficiency, decrease of life time of red blood cells, uremic toxicity, chronic and inflammatory diseases, infections and aluminum toxicity. Severe hyperparathyroidism associated with bone marrow fibrosis and other diseases such as hypothyroidism, hemoglobinopathy may result in anemia (1,3). However, the major cause is decrease of erythropoietin production. Prescription of recombinant erythropoietin (rHu-EPO) is the main step in treatment of anemia in these patients (1,4). It has eliminated the need to frequent blood transfusions and the incidents of infections during the blood transfusion and iron loading and alloantibody production that may cause patient’s sensitivity to donated kidney antigens and complicates kidney transplantation (5).

Aplasia of red blood cells following the prescription of erythropoietin, is a rare but has serious side effects and may be life threatening in CKD (6). Diagnostic criteria for epoetin-induced pure red cell aplasia (PRCA) are treatment with recombinant erythropoietin for at least several weeks, weekly drop hemoglobin of 1 g/dL or transfusion need 1 U per week, reticulocyte below 1%, normal platelets and leukocyte count, skin or systemic reactions, bone marrow with <5% erythroblasts and presence of anti-erythropoietin antibodies in serum, increased percent of transferrin saturation and levels of serum ferritin (1,7).

There are many research reports about the aplasia in kidney patients particularly in European countries (4,8). From 1998 there has been an increase in the number of PRCA that has been caused by epoetin in patients suffering from chronic renal failure and peaking in 2001-2002, however, the number of cases at the present has subsided significantly (9-11).

Different components used to preserve the drug by different manufacturers are assumed the cause of this complication. The method of keeping and transferring the drug in cooling chain is one of the contributing factors in the incident of aplasia. Finally, the method of injection of the drug is also an important factor since the drug is injected subcutaneously (12). One of the main criterion to diagnose this complication is the search of antibody in circulation of the patients (1,3). Despite the fact that bone marrow test of these patients show no erythroblasts, the diagnosis is based on the search for serum antibody to erythropoietin (13).

Treatments include termination of recombinant erythropoietin use, correction of anemia by blood transfusion if necessary, kidney transplantation as the most effective treatment, prescription of cyclosporine A, or cyclophosphamide with corticosteroid to suppress the immune system (14-16).

## 2. Objectives

In the present research, dialysis patients who suffered from anemia despite the consumption of erythropoietin and iron or other necessary compounds, were conducted to bone marrow aspiration and anti-body against erythropoietin. Considering the prevalence of anemia in dialysis patients and its associated complications, timely diagnosis of the cause and its treatment results in improvement of the survival and quality of life of the patient.

## 3. Patients and Methods

In this cross-sectional study 128 patients in the dialysis centers of Kashan were enrolled to the investigation. Data were collected using a questionnaire and a consent form is signed by each patient. The inclusion criteria were being on hemodialysis for at least 3 months, hemoglobin level less than 10 g/dL and use recombinant erythropoietin (rHu-EPO) at least 3 months. The patients examined for anemia through checking their hemoglobin and hematocrit. The quality of dialysis was calculated by KT/V and treated. Then, anemic patients were examined for cause of anemia by checking serum levels of serum iron, ferritin, vitamin B12, folate, thyroid-stimulating hormone (TSH), free T4 and free T3. Additionally bone marrow aspiration was conducted for patients. Exclusion criteria were malignancies, recent blood transfusion, chronic or acute inflammatory or infectious diseases, fever and bleeding. Selected patients, then were tested for serum anti-rHu-EPO antibody level by ELISA method IBL company kit. The sample of anti-rHu-EPO antibody was reported positive or negative.

### 3.1. Ethical issues

1) The research followed the tenets of the Declaration of Helsinki; 2) informed consent was obtained, and they were free to leave the study at any time and 3) this project was approved by ethics committee of Kashan Medical Sciences, Kashan (Ethical code #3294).

### 3.2. Statistical analysis

Following the completion of the research protocol, SPSS 16.0 software was employed to analyze the data. Descriptive statistics as well as t test, analysis of variance (ANOVA) and chi-square test of significance was used to present the results. The cases identified as aplasia of isolated red globulin were examined as case-series and P value less than 0.05 was considered as significant.

## 4. Results

This research was carried out on 128 hemodialysis patients. The result of analysis showed that 55 (43%) patients had hemoglobin level less than 10 mg/dL and 73 (57%) patients had hemoglobin level above 10 mg/dL. In addition, 30 (54.5%) of the patients with anemia were male and 25 (45.5%) were female and 10 (43.4%) patients were under the age 40 years, 16 (48.5%) were between 40 to 60 and 29 (40.3%) were more than 60 years old. Of all dialysis patients, 69 were diabetics while 28 (40.6%) of them were anemic with hemoglobin level less than 10 mg/dL. Around 16 patients suffered from hypertension, while 8 (50%) of them were anemic, one patient suffering from urinary tract obstruction with anemia. Fifteen patients had previous history of glomerulonephritis, while 9 (60%) of them were anemic. In 34 patients, the etiology of end-stage renal disease was not defined, while 13 (38.2%) of them had anemia. In the anemic patients, 3 patients suffered from hypothyroidism and 13 patients had iron deficiency anemia. Remaining anemic patients were examined for anti-erythropoietin antibodies. Around 10 patients were weakly positive for anti-erythropoietin antibody that were not at the acceptable level based on laboratory scale. Only three male patients anti-erythropoietin antibodies were positive and had PRCA.

The result of analysis indicated that 30 (40%) male patients had hemoglobin level less than 10 mg/dL whereas 25 (47.2%) female patients were in this condition. There was no significant association between the gender and the level of hemoglobin (*P*=0.54) (Table 1).

**Table 1:** Distribution of anemic patient dialyzed according to gender in dialysis centers of Kashan*

**Gender**	**Hemoglobin**					
	**<10 g/dL**		**>10 g/dL**		**Total**	
	**Count**	**%**	**Count**	**%**	**Count**	**%**
Male	30	40	45	60	75	58.6
Female	25	47.2	28	52	53	41.4
Total	55	43	73	57	128	100

*P= 0.54.

The result of analysis showed that 10 (43.5%) cases of the patients with hemoglobin level under 10 g/dL were under the age 39 years, 16 (48.5%) were between 39 to 59 and 29 (40.3%) were more than 60 years old. No significant association between age group and anemia was found (Table 2).

**Table 2:** Distribution of anemia in dialysis patients of Kashan dialysis centers according to the age group*

**Age group**	**Hemoglobin level**					
	**<10 g/dL**		**>10 g/dL**		**Total**	
	**Count**	**%**	**Count**	**%**	**Count**	**%**
<39	10	43.5	13	56.5	23	100
40-59	16	48.5	17	51.5	33	100
>60	29	40.3	43	57	72	100
Total	55	43	73	57	128	100

*P= 0.62.

Table 3 shows that 2 (1.6%) patient who were under hemodialysis for one year and the second for five years and received venal erythropoietin injection and 1 patient who was under hemodialysis for two years and received subcutaneous erythropoietin injection had anti-erythropoietin antibodies. No significant association between anti-erythropoietin antibodies and method of injection was found (*P*=0.09). There were no correlation between age, gender, CKD cases, hemodialysis duration, hemoglobin level, rHu-EPO dose and duration and anti-rHu-EPO antibody level.

**Table 3:** Frequency of anti-erythropoietin antibodies according to erythropoietin injection in anemic patients dialyzed in Kashan dialysis centers*

**Antibody**	**Injection method**		**Total**
	**Venal**	**Subcutaneous**	
Negative	122 (98.4%)	3 (75%)	125 (97.6%)
Positive	2 (1.6%)	1 (25%)	3 (2.4%)
Total	124 (100%)	4 (100%)	128 (100%)

*P= 0.09.

## 5. Discussion

In this cross-sectional study 128 patients with chronic kidney failure were examined and 78 (58.6%) of them were male and the rest were female. Kharagjitsingh et al conducted a research including 171 patients receiving dialysis and reported that 115 patients were male (17).

This research revealed that 55 (43%) of the patients had hemoglobin value less than 10 mg/dL and 30 (52.8%) were male whereas 25 (47.2%) were female and 73 (57%) of the patients had hemoglobin value more than 10 mg/dL. In a study conducted by Alqahwaji et al, it was found that the mean hemoglobin value dialysis patient was 8.5 mg/dL and from 22 studied patients, 15 patients had hemoglobin level less than 10 mg/dL (18).

The result of analysis indicated that 69 patients were afflicted to diabetes and 28 (40.6%) of them were anemic (Hgb <10 mg/dL). In addition, no association was found between anemia and diabetes. Masmahisak examined 22 dialysis patients and demonstrated that 35.2% of the patients were afflicted to diabetes (19). In addition, 16 patients were hypertensive that 8 (50%) of them were anemic. Also, 15 patients were suffering from glomerulonephritis that 9 (60%) were anemic. The remaining 34 patients were free from known background disease that 13 (38.2%) were anemic. The study by Masmahisak showed that 45.3% of the patients were hypertensive and 50.1% of the patients were suffering from glomerulonephritis (19).

The majority of studies regarding the treatment of red blood cell aplasia with erythropoietin in dialysis patients are based on case series studies. For example, Jacob et al examined a 58-year-old dialysis patient treated by darbepoetin-alfa (erythropoietin factor). The patient was then afflicted to aplastic anemia (20). In another study, Summers et al described a 81-year-old patient that was suffering from anemic aplasia that after the patient was treated by erythropoietin, antibody was formed in his blood (21).

In the present research anti-erythropoietin antibodies were assessed through ELISA method and were found that in 1/10 density, 10 cases were identified as positive for anti-erythropoietin antibodies which were not in the acceptable level based on laboratory scale and in 1/100 density, only three cases were found having anti-erythropoietin antibody positive level. In patients with positive anti-erythropoietin antibodies, two patients received intravenous erythropoietin and the remained patients received subcutaneous erythropoietin.

Manenti and Vaglio reported only a 72-year-old patient with no background disease who received erythropoietin subcutaneously became PRCA in their dialysis center, while the majority of patients received subcutaneous erythropoietin (22). Accordingly, Vartia et al reported two cases who received subcutaneous erythropoietin injection afflicted to PRCA (23).

Likewise, the study by Casadevall et al, showed that following the first 10 years of treatment only three patients from PRCA were dependent on antibody. They concluded that, the likelihood of antibody formation by epoetin is very low (9).

Furthermore, Shimizu et al reported a rare case of antibody mediated PRCA of a dialysis patient who received intravenous EPO. The diagnosis was based on bone marrow examination and anti-EPO antibody isolation (12). Moreover, Cases et al found, the prevalence of this disease has increased particularly in association with the subcutaneous epoetin (8).

## 6. Conclusions

Based on proportion of patients attending this research, it is clear how each of the injecting method influenced the incidence of aplasia. The result indicated that administration of erythropoietin in patients suffering from kidney failure particularly through the venous route increases the incidence of red blood cell aplasia, however, this increase was not statistically significant and this condition may occur due to any of the two methods venous or subcutaneous injection. There were no correlation between age, gender, CKD cases, hemodialysis duration, hemoglobin level, rHu-EPO dose and duration and anti-rHu-EPO antibody level and PRCA.

## Limitations of the study

The limitation of our study was small sample of study.

## Acknowledgements

We would like to express our appreciation to the full cooperation of the dialysis center staff in collecting the data and staff of research center. This study is adapted from the M.D. thesis of Firoozeh Abdoli (Thesis 29/5/1/612).

## Authors’ contribution

ZC designed the project. FA and MR collected the data. SMMT performed the data analysis. ZC wrote the manuscript. All the authors reviewed and approved the final manuscript.

## Conflicts of interest

The authors declared no competing interests.

## Funding/Support

This article was prepared by support of Research Deputy of medical school of Kashan University of Medical Sciences.
